# Human's Cognitive Ability to Assess Facial Cues from Photographs: A Study of Sexual Selection in the Bolivian Amazon

**DOI:** 10.1371/journal.pone.0011027

**Published:** 2010-06-09

**Authors:** Eduardo A. Undurraga, Dan T. A. Eisenberg, Oyunbileg Magvanjav, Ruoxue Wang, William R. Leonard, Thomas W. McDade, Victoria Reyes-García, Colleen Nyberg, Susan Tanner, Tomás Huanca, TAPS. Bolivia Study Team, Ricardo A. Godoy

**Affiliations:** 1 Heller School for Social Policy and Management, Brandeis University, Waltham, Massachusetts, United States of America; 2 Department of Anthropology, Northwestern University, Evanston, Illinois, United States of America; 3 Department of Psychology, Brandeis University, Waltham, Massachusetts, United States of America; 4 ICREA and Institut de Ciència i Tecnologia Ambientals, Universitat Autònoma de Barcelona, Barcelona, Spain; 5 Department of Anthropology, University of Georgia, Athens, Georgia, United States of America; 6 CBIDSI-Centro Boliviano de Investigación y de Desarrollo Socio Integral, San Borja, Bolivia; 7 Tsimane' Amazonian Panel Study, San Borja, Bolivia; CNRS, France

## Abstract

**Background:**

Evolutionary theory suggests that natural selection favors the evolution of cognitive abilities which allow humans to use facial cues to assess traits of others. The use of facial and somatic cues by humans has been studied mainly in western industrialized countries, leaving unanswered whether results are valid across cultures.

**Methodology/Principal Findings:**

Our objectives were to test (i) if previous finding about raters' ability to get accurate information about an individual by looking at his facial photograph held in low-income non western rural societies and (ii) whether women and men differ in this ability. To answer the questions we did a study during July-August 2007 among the Tsimane', a native Amazonian society of foragers-farmers in Bolivia. We asked 40 females and 40 males 16–25 years of age to rate four traits in 93 facial photographs of other Tsimane' males. The four traits were based on sexual selection theory, and included health, dominance, knowledge, and sociability. The rating scale for each trait ranged from one (least) to four (most). The average rating for each trait was calculated for each individual in the photograph and regressed against objective measures of the trait from the person in the photograph. We found that (i) female Tsimane' raters were able to assess facial cues related to health, dominance, and knowledge and (ii) male Tsimane' raters were able to assess facial cues related to dominance, knowledge, and sociability.

**Conclusions/Significance:**

Our results support the existence of a human ability to identify objective traits from facial cues, as suggested by evolutionary theory.

## Introduction

*For it is true that bones and flesh are not enough to constitute a face, (…) and that is why it is infinitely less physical than the body: it is characterized by the look in the eyes, the expression of the mouth, the wrinkles, by all that conjunction of subtle attributes whereby the souls reveals itself by way of the flesh*

Ernesto Sábato, On Heroes and Tombs. (1961/1981)

Evolutionary theory suggests that natural selection favors the evolution of cognitive abilities which allow humans to use facial and other somatic cues to assess traits of other people, such as sex, age, health, or aggressiveness [1]–[6]. Research suggests that how we judge traits in females and males come from cues associated with reproductive success in ancestral environments [4], [5], [7]–[9]. The ability to judge traits has been studied extensively among non-human animals [10]–[13], but fewer studies have been done among humans, and mostly in affluent industrial societies [5].

To enhance fitness, humans have developed the ability to judge traits in others. As a result selection pressures lead individuals to display these preferred traits. The ability enhances not only biological outcomes, but also social and psychological outcomes. For instance, humans have developed the ability to use facial and somatic cues to assess the ability of others to offer physical protection, access to resources, care for the offspring, and generosity [14]–[20].

Judgments based on physical traits can have long-run effects on the person being judged. For example, compared with people judged as being of average or below-average looks, people judged as beautiful or attractive tend to have higher income and to be more self-confident, receive more attention, and are perceived as being kinder, healthier, or smarter [21]–[24]. The face is a key source of communication and scrutiny because it reveals a wide range of information about the person [25]–[31].

Researchers have demonstrated that raters can judge a person's character by looking at the person's face [32]–[38]. Studies of rater's ability to judge traits from facial features suggest that people can accurately judge reproductive health [7], genetic quality–defined as MHC-heterozygosity [39], [40], sexual orientation [41], [42], aggressiveness [43], [44], physical strength [45], [46], physical fitness[3], intelligence [47], and successful leadership [48]. The ability to interpret facial cues probably reflects past selective pressures to accurately assess other individuals even when one was unable to see the other person's body, as might have happened when clothing or vegetation camouflaged the body [45].

This promising line of research leaves unanswered whether results are valid across cultures. With a few exceptions [34], [45] most of the research known to us come from industrial societies. Studying the ability to judge traits in low-income non-western rural societies matters for two reasons. First, one expects stronger links between people's characteristics and their facial traits are stronger in a poor rural economy. Less healthy environments are more likely to leave an imprint on the body than healthier environments, influencing mate choice [49], [50]. For instance, low-income rural societies are more commonly affected by contagious diseases, high parasite loads, unpredictable food supply [15], [51], [52], and limited resources [14], [53]. Second, if the ability to assess traits is an evolutionary adaptation and does not depend solely on cultural constructs, individuals from low-income non-western rural societies should also be able to accurately assess traits.

Our research aims to explore individuals' ability to assess traits, just by looking at another person's face. Specifically, we examined the ability to assess health, social status (dominance and knowledge), and sociability from the facial photographs of men. We used data from a society of forager-farmers in the Bolivian Amazon, the Tsimane' (pronounced cheeMAU- Nay).

### Hypotheses and rationale

Humans should be able to use facial cues to make accurate inferences about another person's health, social status, and sociability because these traits are associated with reproductive success. We equate social status with dominance and knowledge.

Health: An individual's mate choice is partly determined by the desire for a healthy partner, and it requires identifying facial qualities that signal pathogen resistance and general good health [8], [9], [28], [34], [37], [40]. The immunocompetence-handicap hypothesis states that the immunological system is strained by testosterone, so that producing and maintaining testosterone-dependent ornaments are signs of heritable good health in males [1], [33]. For example, muscle mass, which is generally promoted by testosterone, in energetically expensive to maintain. We expect the nutritional requirements to be higher in more muscular individuals, which make them somewhat less resistant to famines and unpredictable food supply. Sexual dimorphic traits including musculature and enlarged jaws or chins in the case of males, determine mating preferences of females [5], [8], [37]. As we are using only photographs of male faces, our first hypothesis (H_1_) is that female raters should be more accurate than male raters in judging a man's health from a photograph of his face.

Social status: Both female and male raters should be accurate in their assessment of social status from male faces. Social status matters in mate choice because it is associated with access to resources [54], [55], and is also associated with intra-sexual competition, by signaling the capacity to inflict costs on a rival [45]. Among humans, social status tends to come from dominance and “excelling in valued domains” [16]. To operationalize social status we focus on dominance, as the potential use of force and aggression, and on knowledge, as a valued domain that may increase the likelihood of survival.

(i) Dominance: From an evolutionary perspective, dominance results from the use of aggression and potential use of force against sexual rivals [14]. This type of intra-sexual competition is more salient in males than in females [4], [18], [19], [46]. Male faces have several secondary sexual traits that signal dominance [29], [32], [33] and these traits might increase when males face competition [36], [56]. Accurately assessing other's ability to inflict costs (i.e. physical size and muscularity) can be an advantageous way to face competition by discerning whether to avoid or to engage in conflict [18], [45], [57]. Females are also likely to suffer from sexual aggression, such as forced sexual intercourse [4], [14]. Females might have developed the ability to detect physical strength and body size in males; this ability may allow females to shield themselves from potential aggressors and also to select larger mates who can better protect them [45], or provide access to resources (e.g. hunting ability) [58]. Our second hypothesis (H_2_) is that both female and male raters should have the ability of judging a man's dominance from his face.

(ii) Knowledge: An important component of biological fitness in mate choice is the selection of an intelligent mate [59]. Recent research suggests that differences in intelligence might be a consequence of differences in developmental stability, which is reflected through facial symmetry [47]. In industrial societies, general knowledge, schooling, and academic and language skills are associated with better economic outcomes, prestige, and own children's attainments [60]–[62], and the same may be true in small-scale non-western rural societies. For example, previous studies among these societies have found that “modern” human capital (e.g. proficiency in national language, schooling, math skills) improves individual's income [63], and that local knowledge of the environment is related to social status [57], and may increase the likelihood of survival [64], [65]. If mate's intelligence matters in sexual selection and establishing a relationship with prestigious individuals can confer advantages, our third hypothesis (H_3_) is that female raters should be better at judging a man's knowledgeability from his face.

Sociability: Being able to recognize how sociable a person is should influence mate choice, as it enhances life chances by securing access to resources. Cooperation and alliances have been crucial in human evolution [18], [56], [66], and low-income rural societies typically organize in cooperative ways and share resources [57], [67]. There is growing evidence that sociable individuals have priority access to scarce resources [18], [56], [68]. On the other hand, assessing how sociable a man is might also be important for other men. Occasionally humans are likely to turn to allies to gain control over resources, inflicting costs on rivals by forming “aggressive, male bonded coalitions where members support each other in a mutual quest to aggress against each other” [14], [45]. Our fourth hypothesis (H_4_) is that there should be no difference between female and male raters in their ability to judge a man's sociability from his face.

## Materials and Methods

### Tsimane' Amazonian Panel Study (2002–2006)

Tsimane' are a native Amazonian society of forager-farmers who live mostly in the department of Beni, Bolivia [69]. They number about 8,000 individuals, living in about 100 small villages. Data for this article comes from the Tsimane' Amazonian Panel Study (TAPS), which includes all people in 13 Tsimane' villages (1995 people; 332 households). These people have been followed annually since 2002. Several historical and ethnographic studies of the Tsimane' have been published recently [70], [71] and are available at the following web site: www.tsimane.org. The subjects in the photographs were selected within this sample, but the Tsimane' who rated the photographs were selected from outside the 13 villages of the panel study. Because both raters and subjects in the photographs were Tsimane', we asked raters how well they knew the person in the photograph and adjusted for this possible confound when doing the statistical analysis.

### Photographs and ratings

We use color face photographs of 93 Tsimane' males in four age groups: *(a)* 16-20, *(b)* 21–25, *(c)* 26–30, and *(d)* 31–35. We restricted the data collection to male faces because there were too many Tsimane' faces for a judge to rate both the female and the male photographs in a reasonable amount of time, without losing interest in the task. We restrict the age group to 16–35 because these are the years when males and females compete for mates.

The photographs showed only the face of the man against a white background. Men were photographed without hats, necklaces, or eyeglasses, and the photographs were standardized in size and cropped to include the top of the hair, sides and the bottom of the chin. At the bottom of each photograph we included four icons with stones: the icon at the bottom left-most of the photograph had no stones, followed by an icon to the right with one stone, another icon with two stones, and finally an icon with three stones at the bottom right-most of the photograph. The pile with no stones was labeled “not at all”. The pile with one stone was labeled “not very”. The pile with two stones was labeled “a little”, and the pile with three stones was labeled “very”. Each facial photograph was rated for the following perceived traits: healthy, dominant, knowledgeable, sociable, attractiveness, and babyfaceness. We did not use babyfaceness in the analysis because we encountered problems when explaining the concept to raters and therefore we are not sure about the reliability of the measure.

Forty female and 40 male Tsimane' rated the photographs. Half the raters (20 males, 20 females) only evaluated 55 faces, and the other 40 raters (20 males, 20 females) evaluated the remaining 38 faces. The raters indicated their score by pointing to one of four piles of stone at the bottom of the photograph. After rating all the faces in one trait (e.g., healthy), raters were shown the same faces again and asked to rate the face along another traits (e.g., dominance), again using the four piles at the bottom of the photograph. When asking raters to judge traits, we ordered in two ways the sequence in which we asked about the traits. Among some subjects, we first asked about health, followed by dominance, sociability, and ended with knowledge. Among other subjects we first asked about knowledge, followed by sociability, dominance, and ended with questions about health. Surveyors selected at random which of the two sequences to use with a respondent.

A trained Tsimane'-Spanish translator explained the procedure to the raters and assured them of confidentiality. Informed consent was obtained verbally, not in written form, owing to the low levels of literacy in the Tsimane' adult population. The Institutional Review Board for research with human subjects of Brandeis University approved the study protocol and the consent procedure.

### Statistical model and variables

We used the facial photographs as the unit of analysis. For each photograph and for each trait in the photograph, the mean rating by female raters and the mean rating by male raters were obtained by calculating the average values. Thus, for example, the sociability of person “i” was estimated by taking the average rating of sociability for person “i” made by: *(a)* all 20 male raters or *(b)* all 20 female raters. For the statistical analysis, we used the following linear model:



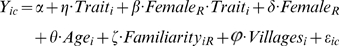
(1)


Equation [1] refers only to one trait (e.g. health). We used a separate multiple regression for each of the four traits: health, dominance, sociability, and knowledge. The subscripts *i, c, and R* stand for the subject *(i)* whose face is shown in the photograph, the objective or self-reported trait *(c)* measured in the panel study, and *(R)* indicates that the variable refers to the rater. To illustrate, *Y_ic_* could be the average health (trait *c* =  health) of man “*i*” as judged by 20 female raters. *Trait_i_* would be a self-reported measure of recent morbidity of the man “*i*” in the photograph. *Female_R_* refers to the raters' sex. *Age_i_* is the self-reported age in years of the person “*i*” shown in the photograph at the time when the photograph was taken (ranges from 15 to 35 years old). *Villages_i_* is a full set of dummy variables for villages, to adjust for the possible effects of village of residence of the man in the photograph. Despite the fact that both the raters and the subjects in the photographs were from different villages, they were all Tsimane', so some raters might have known or heard of the subjects in the photographs. To adjust by this possible confounder, we asked the raters how well they knew the man in the photograph and measured it on a Likert scale. *Familiarity_iR_* refers to how well the raters knew the man “*i*” in the photograph.

Because the outcome variable is the average rating of a trait of the subject either by the 20 female raters or by the 20 male raters, we include two observations for each subject–one observation for the rating by female raters, and one observation for the rating by male raters. Since we had two observations for each subject, we ran the regressions with clustering by subject. For the interaction term, we center the variable *trait_i_* by subtracting its mean value. The Likert-scale points were changed to a scale of 100 to make the results more easily interpretable [30]. *η* represents the effect of male raters, *β* is the differential effect between the score of female raters and male raters (female effect is *η* +*β*), and *δ* represents a shift in the constant in female raters' scores at the mean value of *trait*. Table 1 contains definitions and summary statistics of the variables.

**Table 1:** Definition and summary statistics of variables used in regressions and equation [1].

Name	Definition	Obs.	Mean	Stand. Dev.
***Dependent variables*** a **, ** ***Y_ic_***				
Healthy	Rater's perception of subject's overall health [Likert scale: 1-4, changed to a scale of 100]	182	45.16	9.64
Dominant	Rater's perception of subject's dominant character [Likert scale: 1-4, changed to a scale of 100]	182	31.54	8.04
Knowledgeable	Rater's perception of subject's knowledge [Likert scale: 1-4, changed to a scale of 100]	182	40.99	7.78
Sociable	Rater's perception of subject's sociability [Likert scale: 1-4, changed to a scale of 100]	182	36.48	8.36
***Explanatory variables*** b ***(X_i_)***				
*Trait_i_*				
Ailments (healthy)	# of different ailments of the subject in the two weeks before the day of the interview	91	0.68	0.33
Mid-arm circumference (dominance)	Subjects' mid-arm circumference [cm]	91	25.60	2.92
Ethnobotanical plant knowledge (knowledgeable)	Score of how many of 19 plants read to subject, the subject reported knowing	91	13.23	2.80
Human capital (knowledgeable)	Educational index that includes standardized subject's fluency in Spanish, literacy, and competency in arithmetic (created using principal component analysis)	91	0.04	0.84
Drinks chicha (sociable)	# days the subject drank chicha during the week before the day of the interview	91	0.48	0.70
Somber (sociable)	How did subject feel during interview? [Dichotomous variable, 0: smiled & laughed, guffaw; 1: somber, no laughter]	91	0.54	0.50
*Controls*				
Familiarity_iR_	How well does the rater knows the subject [Likert scale: 1-4, changed to a scale of 100]	91	14.38	9.75
Female_R_	Rater's sex. [Dichotomous variable, 0: male, 1: female]	91	50% fem.	-
Age_i_	Subject's age [years]	91	21.07	5.87
Village_i_	Subject's village [Dichotomous variable] (12 dummies)	13	-	-

*^a^Average across raters by sex (ratings for 91 different Tsimane' subjects)*.

*^b^All explanatory variables were taken as the average for each subject across the 2002–2006 panel.*

*Trait_i_* stands for a vector of traits of the person shown in the photograph. The traits were measured annually during the panel study. We used the mean value of each trait across the five years of the panel to reduce variability from random measurement error.

Health: We asked subjects in the photographs to report the total number of different ailments they had during the two weeks before the interview. We limited the recall period to two weeks before the day of the interview because we found that sickness recalls beyond that period produced unreliable information [72]. A shorter recall period (e.g., one day) might have produced more accuracy, but would have also produced lower variance, and using diaries is impractical due to the high prevalence of functional illiteracy among adults [63].

Dominance: *Mid-arm circumference* of the subjects was measured to the nearest millimeter using plastic tape measures. Mid-arm muscle development is enhanced with greater levels of physical activity. It also reflects muscle development and fat deposition on the upper arm, and proxies for short-run nutritional status [73], [74]. Von Rueden et al. [57] found that physical size among Tsimane' predicted well dyadic fighting ability, which was also related to high status. We use *mid-arm circumference* as a proxy for a person's strength and potential aggression.

Knowledge: Because Tsimane' have varying degrees of exposure to the western society (Rubio et al. 2009), they draw on both traditional and modern forms of knowledge. As a result, we used both ethnobotanical knowledge and modern human capital to measure a person's knowledge. Tsimane' rely on ethnobotanical knowledge for many of their daily activities, including making tools, eating, building houses, or treating common ailments [65], [72], [75]. Previous studies have found a positive association between adult ethnobotanical knowledge and different measures of child health [64] and adult's nutritional status [64], [65]. *Ethnobotanical plant knowledge* is a score that represents the subjects' knowledge of 19 different local wild plants, and we use it as a predictor of knowledgeability. The participants were asked whether they knew each of the plants selected (using the Tsimane' name). The plants included in this measure were the result of 18 months of fieldwork aimed at quantifying the cultural, economic, and practical value of wild plants among Tsimane' [75].

*Modern human capital* is an index created using principal component factor analysis. It includes standardized measures of subject's competence in arithmetic, fluency speaking Spanish, and Spanish literacy (*Chronbach's alpha = 0.77*). Competence in arithmetic and literacy were determined through oral and reading tests and proficiency in spoken Spanish was determined by the interviewer [63].

Sociability: Subject's sociability was measured by how often he drank chicha and whether he was somber during the interviews. Chicha is a traditional beverage fermented from crops such as manioc, plantains, or maize. It is produced at home and distributed by women. As opposed to commercial alcohol, chicha is a cornerstone of Tsimane' social life - it is always consumed in a group social setting, and is the preferred way through which native Amazonians share experiences with each other [76], [77]. *Drinks chicha* stands for the self-reported number of days the person drank chicha the week before the day of the survey. The number of days a person drinks chicha should represent the number of times the person had meaningful social interactions with other villagers. On the other hand, as everyday experience and previous studies suggest, social involvement is strongly associated with smiling [78], [79]. During the interview, surveyors assessed whether the subject (i) was somber, (ii) smiled, (iii) smiled and laughed, or (iv) laughed openly and loudly often. Using this information, we created a dummy variable *somber*, coded as one if the subject was in categories (i) - (ii) and zero otherwise.

Table 2 contains a summary of the Pearson correlations between the ratings of different traits that were assessed by female and male raters from facial photographs of men. The traits measured are all highly significant and positively correlated. Correlation coefficients ranged from a low of 0.51 (knowledgeable-healthy, and sociable-dominant for female raters) to a high of 0.75 (dominant-healthy). The high correlation coefficients between the different traits were expected, as perceived health, dominance, sociability and knowledgeability are related and probably reflect perceived attractiveness [6], [80], [81]. For example, more attractive people might be perceived as healthier, more dominant, more knowledgeable, and as being more sociable than homely people or than people of average attractiveness, and this is due to the halo effect produced by attractiveness. This is further discussed in the robustness analysis.

**Table 2:** Correlation between the ratings of different traits that were assessed by Tsimane' raters from facial photographs of men.

	Healthy	Dominant	Knowledgeable
*(i) Ratings by female raters*			
Dominant	0.745		
Knowledgeable	0.505	0.520	
Sociable	0.540	0.509	0.660
*(ii) Ratings by male raters*			
Dominant	0.746		
Knowledgeable	0.524	0.528	
Sociable	0.711	0.564	0.669
*(iii) Ratings by all raters*			
Dominant	0.745		
Knowledgeable	0.515	0.525	
Sociable	0.610	0.531	0.658

*Note: All correlations significant at p<0.01.*

## Results

Table 3 contains the regression results of the outcome variable–rater's perception of the subject's health, dominance, knowledge, and sociability from the facial photographs–against the explanatory variables of equation [1].

**Table 3:** Regression results for mean ratings of the following perceived traits from facial photographs: healthy [1], dominant [2], knowledgeable [3], and sociable [4].

	Explanatory variables	Healthy	Dominant	Knowledgeable	Sociable
		[1]	[2]	[3]	[4]
Health	[1] Ailments	−0.787			
		(3.145)			
Dominance	[2] Mid-arm circumference		1.361**		
			(0.353)		
Knowledge	[3.1] Ethnobotanical plant knowledge			1.006*	
				(0.415)	
	[3.2] Modern human capital			1.382	
				(1.183)	
Sociability	[4.1] Drinks chicha				1.079
					(1.791)
	[4.2] Somber				−3.587*
					(1.796)
Interaction term^a^	[5.1] Female*trait	−5.611**	−0.344	−0.733*	2.443
		(2.126)	(0.297)	(0.296)	(2.443)
	[5.2] Female*trait^b^			−0.899	1.343
				(1.043)	(2.039)
Controls	[6] Rater's sex (female)	3.197**	1.869*	2.995**	−0.438
		(0.802)	(0.828)	(0.902)	(1.472)
	[7] Subject's age	−0.062	−0.468*	0.385*	−0.146
		(0.165)	(0.195)	(0.176)	(0.131)
	[8] Rater's familiarity with subject	0.286**	0.274**	0.277**	0.295**
		(0.078)	(0.058)	(0.069)	(0.069)
	Observations	182	182	130	156
	R^2^	0.172	0.257	0.370	0.323
F-test: Female & interaction term		9.61**	2.93	6.13** c	0.43^c^

*Note:(1) All regressions include a full set of village dummy variables, clustering by subject, and robust standard errors in parenthesis.*

**p<0.01, * p<0.05.

*^a^Trait is centered in the interaction term.*

*^b^The traits in the interaction term [5.2] are modern human capital in column [3] and somber in column [4].*

*^c^F-test of the two traits and the two interaction terms.*

The results shown in column [1], row [5.1], Table 3 suggest a significant difference between the way females and males rated the health status of the subject in the photographs. On average, each additional ailment reported by the subject in the photograph was associated with the subject being rated as having 5.61 percentage-points worse health by female raters than by male raters (*p = 0.01*). We also ran separate regressions for female and male raters (not shown). When including only female raters, an additional ailment reported by the subject in the photograph was associated with a decrease of 6.72 percentage-points in health assessment (*p = .026)*. We did not find statistical evidence of male rating corresponding to the number of ailments experienced by the man in the photograph (η = −0.47, p = 0.889).

Mid-arm circumference (Table 3, column [2], row [2]) was positively associated with rater's perceptions of the subjects' dominant character. On average, a one cm increase in the subject's mid-arm circumference was associated with the subject being perceived 1.36 percentage-points more dominant by both female and male raters (*p<0.001*). This finding might be better understood with an example. The average man in the sample had a mid-arm circumference of 25.60 cm. If this man saw an increase of 10% in his mid-arm circumference, raters would increase their average evaluation of his dominance from his facial photograph by 3.46 percentage-points. To be sure about the result, we did separate regressions for female and male raters. Those regressions are not shown, but they suggest that the results held true for female and male raters (females: *η = 1.233, p = 0.004*; males: *η = 1.145, p = 0.005*). Row [5.1] suggests no significant difference in the ability of female and male raters to judge differences in men's dominance from their facial photograph. However, female raters rated dominance 1.87 percentage-points higher than male raters at the average mid-arm circumference in the sample *(p = 0.026)* (row [6]).

We obtained ambiguous results regarding raters' ability to assess how knowledgeable the subjects in the photograph were (Table 3, column [3]). We found a positive and significant association between the ethnobotanical plant knowledge of a man and the rating he received in how knowledgeable he was (row [3.1]). On average, a one-point increase in the test score of ethnobotanical plant knowledge was associated with an increase of 1.01 percentage-points in the male raters' score of knowledgeability (*p = 0.018*), and 0.27 percentage-points in female raters score (row [3.1] + row[5.1]). We found no evidence that modern human capital was associated with ratings of knowledgeability (rows [3.2], [5.2]). On average, females rated an average man in the photographs as being *2.995* percentage-points more knowledgeable than male raters *(p = 0.012)* (row [6]).

We found no association between the number of days men drank chicha and how sociable they were judged by raters (Table 3, column [4], row [4.1]). However, the results suggested a negative and statistically significant association between how somber the subject was during the interviews and how sociable he was judged by raters (row [4.2]). We ran separate regressions for female and male raters' judgment of how sociable the men in the photographs were. Those regressions are not shown, but they suggest that only males' assessment of sociability corresponded to the somberness of the subject (females: *η = −2.071, p = 0.284*; males: *η = −3.747, p = 0.039*). Our results suggest that, controlling for chicha drinking, a man who was somber during the interviews was on average perceived by male raters as 3.75 percentage-points less sociable than a man who had open displays of mirth during the interviews.

Although used only as a control, the results on row [8], Table 3 suggest that how well the rater knew the man in the photograph was on average significantly associated to her or his perception of the man's health, dominance, knowledgeability, and sociability. Recall that both raters and the men in the photographs were Tsimane', so in some cases the rater was familiar with the man in the photograph. If so, on average, the rater tended to rate the man in the photograph as being more healthy, dominant, knowledgeable, and sociable than if the rater was not familiar with the man in the photograph. We discuss this further in the limitations section.

### Robustness analysis

To assess the robustness of the main regression results we use different measures for health, dominance, and sociability. We used principal component factor analysis to create two factors, *illness* as a proxy for subjects' health and *formidability* as a proxy for physical body size and strength. The *illness* factor includes three standardized measures of self-reported illness: bed ridden days, number of days the person was sick, and total number of different ailments the person had during the 14 days before the day of the interview (*Chronbach's alpha = 0.74)*. The dominance factor, *formidability,* includes standardized measurements of subjects' fat-free mass, standing physical stature (*height*), and mid-arm circumference (*Chronbach's alpha = 0.94)*. Fat-free mass was estimated using the subject's weight [kg] minus his total body fat [kg]. Subjects were weighed without shoes and hats, and their total body fat was determined using bioelectrical impedance analysis (BIA). Finally, we used a persons' *ability to borrow* 100 bolivianos (∼US$15) in an emergency as an alternative proxy for sociability, or effectiveness of social network to cope with emergencies.

Table 4 contains the results of the robustness analysis. For brevity, in Table 4 we only report the coefficients of the new measures and the coefficients of the new interaction terms (*η* and *β* in Eq.1).

**Table 4:** Robustness analysis of the results using other measures of traits.

Dependent variable	Alternative trait	[1] Trait	[2] Trait*female	[3] *F-test* a
[1] Healthy	Illness^i^	−0.439	−1.985*	8.67
		(1.256)	(0.792)	(0.001)
[2] Dominant	Formidability^ii^	3.480*	−0.728	2.94
		(1.438)	(1.098)	(0.058)
[3] Sociable	Ability to borrow	2.255	0.021	0.14^b^
		(1.680)	(2.121)	(0.934)
	Somber	−3.803*	1.194	
		(1.774)	(2.055)	

*Note: same as Table 3.*

*^a^F-test: female & interaction term (p value in parentheses).*

*^b^F-test of the two traits and the two interaction terms.*

*^i^Bed-ridden days, days ill, number of ailments. Chronbach's alpha = 0.742; Eigenvalues: factor 1 = 1.994 (66% of total variance), factor 2 = 0.718.*

*^ii^Mid-arm circumference, height, fat-free mass. Chronbach's alpha = 0.944; Eigenvalues: factor 1  = 2.675 (89% of total variance), factor 2 = 0.269.*

The results of the additional analysis support the main results. For example, health measured using an illness factor (Table 4, column [1], row [1]) continued to bear a negative association with rater's health assessment, and again, was not statistically significant (*p = 0.728*). We also found a significant difference between the ability of female and male raters to assess the health status of the subject in the photographs (p = 0.014) (row [1], column [2]). We ran separate regressions for female and male raters. Results suggest that a one standard deviation increase in *illness* was associated with a decrease of 2.42 percentage-points in health assessment by female raters (p = 0.060), but we did not find a significant result for male raters (η = −0.395, p = 0.766).

Dominance measured using *formidability* also continued to bear a positive association with rater's assessment of a man's dominance. A one standard deviation increase in formidability was associated with a 3.48 percentage-points increase in rating of how dominant the man in the photograph was (p = 0.018). We found no difference between female and male raters in their ability to assess a man's dominance from a photograph of his face (Table 4, columns [1], [2], row [2]). The results for sociability also held up. How somber the man in the photograph was during the interviews bore a negative and statistically significant relation with how sociable he was judged by raters (p = 0.035). We found no evidence that the man's ability to borrow was associated with ratings of sociability (Table 4, column [1], [2], row [3]). We ran separate regressions for female and male raters (not shown), and found that only male raters' judgment of how sociable the man in photographs corresponded to his somberness. On average, a man who was somber during the interviews was perceived as 3.80 percentage points less sociable than a man who laughed and guffawed (p = 0.028).

Recall from the earlier discussion that perceptions of traits could be influenced by a halo effect of perceived attractiveness of the person evaluated. We tested if attractiveness was mediating the effect of perceived traits. For each of the traits studied, we performed a Sobel test with bias-corrected bootstrap confidence intervals (200 replications) including the same covariates as in Eq.1 to test if there was a halo effect of attractiveness. The only significant mediation effect we found was for *drinks chicha* (η = 2.12, bootstrap standard error  = 0.867). The results suggest that the halo effect of attractiveness was not significant in the raters' perception of the traits studied. This lack of significance might be because attractiveness effect is less important when using male subjects [82], [83], but we do not have data to test this hypothesis.

## Discussion

Our findings suggest that the ability to accurately assess traits associated with sexual selection holds across cultures. We found support for our hypothesis that female raters are more accurate than male raters in judging other men's health status by looking at his face (H_1_). We also found evidence suggesting that female and male raters were able to accurately assess a man's dominance (H_2_). We expected that female raters would be more accurate than male raters in assessing knowledgeability from a man's photograph (H_3_), and we found that both were accurate. Finally, we found partial support to our hypothesis that female and male raters could assess how sociable a man was by looking at his facial photograph (H_4_). Our analysis suggests that only male raters were accurate.

Female raters in the sample were able to accurately predict health status in other males, but we found no evidence of this ability in male raters. As suggested by sexual selection theory, female's cognitive specialization in identifying healthy mates relates to successful mating and increases the likelihood of offspring survival. Another explanation might be that the difference results from gender roles in low-income, rural societies, where women act as health-care managers [84], [85]. For example, using ethnographic data and household surveys (n = 153) from peri-urban communities in the western Amazon, Wayland [86] found that women's role as health-care managers enhanced their skills at judging health. Similarly, McDade et al. [64] found that child health among the Tsimane' was strongly associated to maternal ethnobotanical knowledge, but had a weaker association with paternal ethnobotanical knowledge.

A man's mid-arm circumference had a significant positive association with a rater's perception of the man's dominance. This finding is consistent with evolutionary theory because both female and male raters identified cues indicating physical size or upper-body strength. Male's ability to assess dominance might be a result of intra-sexual competition [18], [57]. Females' ability to assess dominance might indicate the need to determine the risk of potential aggression [4], [14], desirable physical size, muscularity [45], or hunting ability [58]. We found no evidence that female and male raters differ in their ability to assess a man's dominance from his facial cues. This findings are particularly interesting, as a naïve prediction based upon cultural/linguistic evidence among the Tsimane', who are a relatively egalitarian society [87], might lead one to believe that dominance is not an important construct. However, our findings suggest that despite cultural mechanisms to enforce and reflect social norms of egalitarianism, a man with bigger mid-arm circumference and greater *formidability* was systematically rated as more dominant by female and male raters. Sell et al. [45] found similar results on the ability of college students from the United States to assess upper body strength of men of different cultures just by looking at their facial photographs. The results we obtained using raters from a low-income non-western rural society suggest that the ability to assess dominance (i.e. potential use of force and aggression) of men from their faces is an evolutionary adaptation.

The ethnobotanical plant knowledge of the men in the photographs was positively associated with both female and male raters' perception of how knowledgeable the men were. Plant knowledge is a valuable domain among Tsimane', as it is related to enhanced own and child well-being. We found no association between a man's modern human capital and the raters' perception of how knowledgeable he was. This stark difference between the ability of raters to assess traditional Tsimane' knowledge and modern human capital might be explained by the differences in social esteem derived from traditional knowledge and modern human capital among the Tsimane'. The Tsimane' villages are located along the Maniqui river, and vary in distance from the town of San Borja (population ∼19,000) with distances ranging from less than an hour walking, up to several weeks. There are varying degrees of acculturation among the Tsimane' [88], and the acquisition of modern human capital is probably more related to the village where the individual lives than to his intellectual ability. Conversely, more traditional skills such as horticultural and plant knowledge are commonly used to treat ailments and manage fires, activities which are valued among Tsimane' [70]. Traditional knowledge is more likely to confer esteem and respect [57]. Other types of traditional knowledge, such as knowledge of hunting [58], tool manufacture, or shamanic competence [57] might also be valued in a foraging and hunting society. On average, female raters tended to rate knowledge higher than male raters. However, a one-point increase in the man's test score of ethnobotanical plant knowledge was associated with a greater increase in male raters' perception of how knowledgeable the man in the photograph was than in female raters' perception. The higher average rating of female raters and their smaller response to changes in a man's actual knowledge might be a result of gender roles; however, we lack data to explore the topic because we only have photographs of male Tsimane'. Thus, we have no convincing explanation for the finding.

Finally, only male raters in our sample were able to accurately assess sociability from the facial cues of the men in the photographs. We expected both female and male raters to be able to accurately assess sociability from the photographs because cooperation and alliances have mattered for survival [18], [56] and because assessing another person's sociability might enhance life chances. However, we found no evidence that female raters were able to accurately judge the sociability of the man in the photograph. This finding suggests that sociability might be a characteristic more relevant to male raters. A plausible explanation for why men have greater ability to assess sociability has to do with intra-sexual male competition. Males might need this ability because they have to evaluate (i) aggression against a sexual rival or (ii) the potential aggression of allies. The assessment of potential aggression due to intra-sexual competition in males is further supported by our findings suggesting that male raters were also able to accurately assess dominance and knowledge from the photographs of other men's faces, and that all males in the survey are within active mating years.

### Limitations

The Tsimane' are a highly endogamous society practicing preferential cross-cousin marriage. Consequently, most Tsimane' are linked by kinship bonds of blood and marriage. In such an inward-looking, closely-knit, small-scale society it is possible that people learn the facial cues and traits of some families or ‘lineages’. Since facial features are substantially heritable [89], [90], and the other traits of interest likely also tend to cluster along familial lines for both genetic and cultural reasons, facial characteristics might be accurate markers for other traits. When asking a Tsimane' to rate a person who resembles someone from group X, the rater may have drawn on a common pool of cultural knowledge to judge X. The same experiment carried out in a larger more exogamous society might produce different results owing to less distinct and well known familial facial characteristics.

The results might also be affected by the use of photographs instead of direct facial assessment. The use of photographs may be producing smaller effects because facial assessment requires coding at multiple levels [25]. Some facial traits might be partially obscured in photographs. For example, cognitive specializations to assess the traits studied might rely not only on the shape and the relative position of a person's facial features, but also on facial expressions, or on the look in the eyes, and perhaps even on smell. The use of photographs and printed material is rare in a largely pre-literate society such as the Tsimane', a shortcoming of the study that probably makes our findings conservative since people in real life will make decisions based on much better cues.

### Conclusions

The findings in this study provide support for the hypothesis that the propensity to accurately judge people's traits based on facial features is an evolutionary specialization that probably reflects past selective pressures on mating choices and competition. Both male and female native Amazonian raters were able to evaluate a man's social status, as defined by dominance and knowledge, just by looking at a photograph of his face, using much poorer information than available in reality. Additionally, men raters were able to accurately assess sociability, and female raters were able to assess health status from facial photographs of men in the prime of their mating years.

Male native Amazonian raters were able to assess another man's knowledge, dominance, and sociability by looking at a photograph of his face, but they were not able to rate the health status of the man in the photograph. This finding hints at the idea that male raters are assessing the potential use of aggression of other males due to intra-sexual competition, which is likely to result from their physical size, relative ability, prestige, and potential allies. Female native Amazonian raters were able to assess a man's health, knowledge, and dominance from a photograph of his face, but could not judge sociability. This finding suggests that females may enhance fitness by assessing pathogen resistance, ability, physical strength and formidability in a mate, as predicted by sexual selection theory. Females tended to rate the men in the photographs as having more knowledge than male raters, and this might be caused by differences in social status within Tsimane' society, but, we do not have enough information to test this interpretation. Overall, our findings provide evidence that our cognitive abilities seem to be ancestrally determined, as suggested by evolutionary theory.

## References

[1] Folstad I, Karter A (1992). Parasites, bright males and the immunocompetence handicap.. The American Naturalist.

[2] Getty T (2002). Health versus Parasites.. The American Naturalist.

[3] Honekopp J, Bartholome T, Jansen G (2004). Facial Attractiveness, Symmetry and Physical Fitness in Young Women.. Human Nature.

[4] Jones D, Brace C, Jankowiak W, Laland K, Musselman L (1995). Sexual-Selection, Physical Attractiveness, and Facial Neoteny: Cross-cultural Evidence and Implications (and Comments and Reply).. Current Anthropology.

[5] Weeden J, Sabini J (2005). Physical Attractiveness and Health in Western Societies: A Review.. Psychological Bulletin.

[6] Zebrowitz L, Hall J, Murphy N, Rhodes G (2002). Looking Smart and Looking Good: Facial Cues to Intelligence and their Origins.. Personality and Social Psychology Bulletin.

[7] Smith M, Perrett D, Jones B, Cornwell R, Moore F (2006). Facial appearance is a cue to oestrogen levels in women.. Proceedings of the Royal Society.

[8] Grammer K, Fink B, Moller A, Thornhill R (2003). Darwinian aesthetics: Sexual-selection and the biology of beauty.. Biological Reviews.

[9] Hill K, Hurtado M (1996). The Ache Life History.. The Ecology and Demography of a Foraging People.

[10] Andersson M, Simmons L (2006). Sexual-selection and mate choice.. Trends in Ecology & Evolution.

[11] Dawkins M, Guilford T (1991). The corruption of honest signaling.. Animal Behavior.

[12] Endler J (1992). Signals, Signal Conditions, and the Direction of Evolution.. The American Naturalist.

[13] Endler J, Basolo A (1998). Sensory ecology, receiver biases and sexual-selection.. Trends in Ecology & Evolution.

[14] Buss D, Duntley J, Schaller M, Simpson JA, Kenrick DT (2006). The Evolution of Aggression.. Evolution and Social Psychology.

[15] Gangestad SW, Scheyd GJ (2005). The Evolution of Human Physical Attractiveness.. Annual Review of Anthropology.

[16] Henrich J, Gil-White F (2001). The evolution of prestige. Freely conferred deference as a mechanism for enhancing the benefits of cultural transmission.. Evolution and Human Behavior.

[17] Mazur A, Halpern C, Udry J (1994). Dominant looking male teenagers copulate earlier.. Ethology and Sociobiology.

[18] Sundie J, Cialdini R, Griskevicius V, Kenrick D, Schaller M, Simpson J, Kenrick D (2006). Evolutionary Social Influence.. Evolution and Social Psychology.

[19] Taylor S, Gonzaga G, Schaller M, Simpson J, Kenrick D (2009). Evolution, Relationships, and Health: The Social Shaping Hypothesis.. Evolution and Social Psychology.

[20] Masur A, Halpern C, Udry J (1994). Dominant looking male teenagers copulate earlier.. Ethology and Sociobiology.

[21] Anderson S, Adams G, Plaut V (2008). The Cultural Grounding of Personal Relationship: The Importance of Attractiveness in Everyday Life.. Journal of Personality and Social Psychology.

[22] Biddle J, Hamermesh D (1998). Beauty, Productivity and Discrimination: Lawyer's Looks and Lucre.. Journal of Labor Economics.

[23] Hamermesh D, Biddle J (1994). Beauty and the Labor Market.. The American Economic Review.

[24] Langlois J, Kalakanis L, Rubenstein A, Larson A, Hallam M (2000). Maxims or Myths of Beauty? A Meta-analytic and Theoretical Review.. Psychological Bulletin.

[25] Bruce V, Young A (1986). Understanding face recognition.. British Journal of Psychology.

[26] Hassin R, Trope Y (2000). Facing faces: studies on the cognitive aspects of physiognomy.. Journal of Personality and Social Psychology.

[27] Hess U, Adams R, Kleck R, Or J (2008). The devil is in the details - the meanings of faces and how they influence the meanings of facial expressions.. Affective Computing: Focus on emotion expression, synthesis, and recognition: I-techonline/I-Tech.

[28] Reis V, Zaidel D (2001). Brain and Face: Communicating Signals of Health in the Left and Right Sides of the Face.. Brain and Cognition.

[29] Swaddle J, Reierson G (2002). Testosterone increases perceived dominance but not attractiveness in human males.. Proceedings of the Royal Society.

[30] Zaidel D, Aarde S, Baig K (2005). Appearance of symmetry, beauty, and health in human faces.. Brain and Cognition.

[31] Little AC, Jones BC, Waitt C, Tiddeman BP, Feinberg DR (2008). Symmetry Is Related to Sexual Dimorphism in Faces: Data Across Culture and Species.. Plos One.

[32] Carré J, McCormick C (2008). In your face: facial metrics predict aggressive behavior in the laboratory and in varsity and professional hockey players.. Proceedings of the Royal Society.

[33] Grammer K, Thornhill R (1994). Human (Homo sapiens) facial attractiveness and sexual-selection: The role of symmetry and averageness.. Journal of Comparative Psychology.

[34] Jones D, Hill K (1993). Criteria of facial attractiveness in five populations.. Human Nature.

[35] Kalick S, Zebrowitz L, Langlois J, Johnson R (1998). Does Human Facial Attractiveness Honestly Advertise Health? Longitudinal Data on an Evolutionary Question.. Psychological Science.

[36] Pound N, Penton-Voak I, Surridge A (2009). Testosterone responses to competition in men are related to facial masculinity.. Proceedings of the Royal Society Series B.

[37] Rhodes G, Yoshikawa S, Palermo R, Simmons L, Peters M (2007). Perceived health contributes to the attractiveness of facial symmetry, averageness, and sexual dimorphism.. Perception.

[38] Zebrowitz L, Rhodes G (2004). Sensitivity to “bad genes” and the anomalous face overgeneralization effect: cue validity, cue utilization, and accuracy in judging intelligence and health.. Journal of Nonverbal Behavior.

[39] Lie H, Rhodes G, Simmons L (2008). Genetic Diversity Revealed in Human Faces.. Evolution.

[40] Roberts S, Little A, Gosling L, Perret D, Carter V (2005). MHC-heterozygosity and human facial attractiveness.. Evolution and Human Behavior.

[41] Rule NO, Ambady N, Adams RB, Macrae CN (2008). Accuracy and Awareness in the Perception and Categorization of Male Sexual Orientation.. Journal of Personality and Social Psychology.

[42] Rule NO, Ambady N, Hallett KC (2009). Female sexual orientation is perceived accurately, rapidly, and automatically from the face and its features.. Journal of Experimental Social Psychology.

[43] Carre JM, McCormick CM, Mondloch CJ (2009). Facial Structure Is a Reliable Cue of Aggressive Behavior.. Psychological Science.

[44] Carre JM, McCormick CM (2008). In your face: facial metrics predict aggressive behaviour in the laboratory and in varsity and professional hockey players.. Proceedings of the Royal Society B-Biological Sciences.

[45] Sell A, Cosmides L, Tobby J, Sznycer D, Von Rueden C (2009). Human Adaptations for the Visual Assesment of Strenght and Fighting Ability from the Body and the Face.. Proceedings of the Royal Society Series B.

[46] Fink B, Neave N, Seydel H (2006). Male Facial Appearance Signals Physical Strength to Women.. American Journal of Human Biology.

[47] Zebrowitz LA, Rhodes G (2004). Sensitivity to “bad genes” and the anomalous face overgeneralization effect: Cue validity, cue utilization, and accuracy in judging intelligence and health.. Journal of Nonverbal Behavior.

[48] Rule NO, Ambady N (2009). She's Got the Look: Inferences from Female Chief Executive Officers' Faces Predict their Success.. Sex Roles.

[49] Little AC, Apicella CL, Marlowe FW (2007). Preferences for symmetry in human faces in two cultures: data from the UK and the Hadza, an isolated group of hunter-gatherers.. Proceedings of the Royal Society B-Biological Sciences.

[50] DeBruine LM, Jones BC, Crawford JR, Welling LLM, Little AC (2010). The health of a nation predicts their mate preferences: cross-cultural variation in women's preferences for masculinized male faces.. Proceedings of The Royal Society.

[51] Grammer K, Fink B, Moller A, Manning J (2005). Physical Attractiveness and Health: Comment on Weeden and Sabini (2005).. Psychological Bulletin.

[52] Gurven M, Kaplan H (2007). Longevity among Hunter-Gatherers: A Cross-Cultural Examination.. Population and Development Review.

[53] Durham WH (1976). Resource Competition and Human Aggression, Part I: A Review of Primitive War.. The Quarterly Review of Biology.

[54] Reyes-García V, Molina J, Broesch J, Calvet L, Huanca T (2008). Do the aged and knowledgeable men enjoy more prestige? A test of predictions from the prestige-bias model of cultural transmission.. Evolution and Human Behavior.

[55] Buss DM (2007). The Evolution of Human Mating.. Acta Psychologica Sinica.

[56] Taylor S, Gonzaga G, Schaller M, Simpson J, Kenrick D (2006). Evolution, Relationships, and Health: The Social Shaping Hypothesis.. Evolution and Social Psychology.

[57] Von Rueden C, Gurven M, Kaplan H (2008). The multiple dimensions of male social status in an Amazonian society.. Evolution and Human Behavior.

[58] Gurven M, Winking J, Kaplan H, Von Rueden C, McAllister L (2009). A Bioeconomic Approach to Marriage and the Sexual Division of Labor.. Human Nature.

[59] Miller GF, Bock G, Goode J, Webb K (2000). Sexual selection for indicators of intelligence.. The Nature of Intelligence: John Wiley.

[60] Wolfe B, Haveman R, Helliwell JF (2001). Accounting for the Social and Non-Market Benefits of Education.. The Contribution of Human and Social Capital To Sustained Economic Growth and Well-Being: International Symposium Report,.

[61] Mincer J (1958). Investment in Human Capital and Personal Income Distribution.. The Journal of Political Economy.

[62] Weber M (1947). The Theory of Social and Economic Organization..

[63] Godoy R, Karlan D, Rabindran S, Huanca T (2005). Do modern forms of human capital matter in primitive economies? Comparative evidence from Bolivia.. Economics of Education Review.

[64] McDade T, Reyes-García V, Blackington P, Tanner S, Huanca T (2006). Ethnobotanical knowledge is associated with indices of child health in the Bolivian Amazon.. Proceedings of National Academy of Sciences.

[65] Reyes-García V, McDade TW, Vadez V, Huanca T, Leonard W (2008). Non-market Returns to Traditional Human Capital: Nutritional Status and Traditional Knowledge in a Native Amazonian Society.. Journal of Development Studies.

[66] Sober E, Wilson DS (1998). Unto others: the evolution and psychology of unselfish behavior..

[67] Gil DG, Theory, Analysis (1992). Unravelling Social Policy.. and Political Action Towards Social Equality.

[68] Brabec M, Godoy RA, Reyes-García V, Leonard WR (2007). BMI, Income, and Social Capital in a Native Amazonian Society: Interaction Between Relative and Community Variables.. American Journal of Human Biology.

[69] Leonard W, Godoy R (2008). Tsimane' Amazonian Panel Study [TAPS].. Economics and Human Biology.

[70] Huanca T (2008). Tsimane' oral tradition, landscape, and identity in tropical forest..

[71] Martinez-Rodriguez MR (2009). Ethnobotanical Knowledge Acquisition Among Tsimane' Children in the Bolivian Amazon [Dissertation]: University of Georgia.

[72] Byron E (2003). Market Integration and Health: The Impact of Markets on the Nutritional Status, Morbidity, and Diet of the Tsimane' Amerindians of Lowland Bolivia..

[73] Foster Z, Byron E, Reyes-García V, Huanca T, Vadez V (2005). Physical Growth and Nutritional Status of Tsimane' Amerindian Children of Lowland Bolivia.. American Journal of Physical Anthropology.

[74] Frisancho A (1990). Anthropometric Standards for the Assessment of Growth and Nutritional Status..

[75] Reyes-García V, Huanca T, Vadez V, Leonard W, Wilkie D (2006). Cultural, practical, and economic value of wild plants: a quantitative study in the Bolivian Amazon.. Economic Botany.

[76] Godoy R, Reyes-García V, McDade T, Huanca T, Leonard WR (2006). Does Village inequality in modern income harm the psyche? Anger, fear, sadness and alcohol consumption in a pre-industrial society.. Social Science & Medicine.

[77] Overing J, Passes A (2007). The anthropology of love and anger.. The aesthetics of conviviality in native Amazonia.

[78] Kraut RE, Johnston RE (1979). Social and Emotional Messages of Smiling: An Ethological Approach.. Journal of Personality and Social Psychology.

[79] Hess U, Beaupré MG, Cheung N, Abel M, Ceia C (2002). Who to whom and why – cultural differences and similarities in the function of smiles.. An empirical reflection on the smile.

[80] Folland S (2007). Does “community social capital” contribute to population health?. Social Science and Medicine.

[81] Kumagai Y, Morioka I, Yoshimasu K, Tomita H, Miyai N (2008). Relationships of Self-Reported Physical Health, Sociability, and Spiritual Life with Mental Health: An Investigation According to Gender and Life Stage.. Nippon Eiseigaku Zasshi (Japanese Journal of Hygiene).

[82] Kaplan RM (1978). Is Beauty Talent? Sex Interaction in the Attractiveness Halo Effect.. Sex Roles.

[83] Lucker GW, Beane WE, Helmreich RL (1981). The Strength of the Halo Effect in Physical Attractiveness Research.. Journal of Psychology.

[84] Arnold D, Grewal I, Kaplan C (2006). Women and Medicine.. An Introduction to Women's Studies, Gender in a Transnational World.

[85] Berman P, Kendall C, Bhattacharyya K, Sirageldin I, Mosley H (1988). The Household Production of Health: Putting People at the Center of Health Improvement.. Toward More Efficacy in Child Survival Strategies.

[86] Wayland C (2001). Gendering Local Knowledge: Medicinal Plant Use and Primary Health Care in the Amazon.. Medical Anthropology Quarterly.

[87] Godoy RA, Gurven M, Byron E, Reyes-García V, Keough J (2004). Do Markets Worsen Economic Inequalities? Kuznets in the Bush.. Human Ecology.

[88] Rubio K, Undurraga EA, Magvanjav O, Gravlee C, Huanca T (2009). Modernization and culture loss: A natural experiment among native Amazonians in Bolivia.. TAPS Working Paper.

[89] Lundstrom A, McWilliam J (1988). Comparison of Some Cephalometric Distances and Corresponding Facial Proportions with Regard to Heritability. European Journal of Orthodontics.

[90] Carels C, Cauwenberghe NV, Savoye I, Willems G, Loos R (2001). A quantitative genetic study of cephalometric variables in twins.. Clinical Orthodontics and Research.

